# Modeling and Verification of an Acquisition Strategy for Wheel Loader’s Working Trajectories and Resistance

**DOI:** 10.3390/s22165993

**Published:** 2022-08-11

**Authors:** Shaojie Wang, Yue Yin, Yanfeng Wu, Liang Hou

**Affiliations:** 1 Department of Mechanical and Electrical Engineering, Xiamen University, Xiamen 361102, China; 2 Shenzhen Research Institute of Xiamen University, Shenzhen 518057, China

**Keywords:** wheel loader, working resistance, working trajectory, data collection

## Abstract

To overcome the difficulty of collecting the working resistance and working trajectory of a wheel loader, this paper constructs a statics model of the bucket working resistance and a kinematics model of the working trajectory in the shoveling process and analyzes the key parameters of measuring the working resistance and working trajectory. Based on this, a working resistance and working trajectory acquisition strategy is proposed. To verify the effectiveness of the acquisition strategy, the in-service operation data of fine sand and loose soil shoveled by the wheel loader are collected and analyzed. Then, the test-fitted working resistance and working trajectory are obtained, and the working trajectory is input into the RecurDyn–EDEM co-simulation model to obtain the simulation-fitted working resistance. Considering the complex working conditions of the wheel loader, it is difficult to obtain accurate working resistance, and the actual working resistance is also a relative value. Therefore, a strong correlation between the two curves indicates that the acquisition strategy of the wheel loader’s working trajectory and working resistance proposed in this paper is feasible.

## 1. Introduction

Working resistance is a key parameter of the power transmission system of a wheel loader, and its value is closely related to the working trajectory. The working resistance and trajectory of a wheel loader affect the control accuracy of the shoveling trajectory. Thus, the collection of working resistance and trajectory is essential to realizing intelligent control of the shoveling trajectory of the wheel loader.

In the research field of wheel loader’s working trajectory, Zhao Tengyun et al. [1] analyzed the bucket shoveling process and working trajectory and explored the relevant parameters of the bucket shoveling trajectory. R. Filla et al. [2] simulated multiple shoveling trajectories by Pasimodo and compared various filling strategies by building a loader bucket model. Chen YanHui et al. [3] studied different bucket processes by using discrete element theory and then found a new operating trajectory with the least bucket resistance. Yu Xiangjun [4] used co-simulation by RecurDyn and EDEM to optimize the shoveling trajectory based on three methods including direct retraction, slicing, and segmentation. Yu Meng et al. [5] proposed an optimal planning scheme of the bucket trajectory in the LHD (Load–Haul–Dump) automatic shoveling system to improve the effectiveness of the scooping operation. In terms of trajectory planning and control, a data-driven approach is becoming mainstream. Osher A et al. [6] studied wheel loader trajectory planning and control based on different algorithms such as deep reinforcement learning. Zhao Pengbing et al. [7], provided general ideas and methods for designing hydraulic driving transmission and control systems by studying the design of hydrostatic driving systems and control algorithms for loaders. Shi Junren et al. [8] planned and predicted loader obstacle avoidance trajectories using different algorithms. Hong BeiChuan et al. [9] studied the optimal control of the wheel loader operation process based on different optimization methods such as approximate dynamic programming. In contrast, Frank B et al. [10] searched for the optimal path considering fuel efficiency and environmental impacts. S Sarata et al. [11] proposed a method of path generation for wheel loaders by a stereo-vision system and the scooping direction that imposed the least moment on the bucket as the scooping position.

In the research field of wheel loader working resistance, Chen Lele [12] proposed that the work resistance can be divided into horizontal resistance and vertical resistance and formulated a theoretical calculation formula of the limit value. Huang Pengpeng et al. [13] studied the influencing factors of working resistance by using discrete element theory. Li Ru et al. [14,15,16] conducted a simulation study on the working resistance of various materials based on EDEM. Through the analysis of the working resistance of the wheel loader, Cao BingWei et al. [17] theoretically proved the feasibility of the energy-saving operation of the wheel loader. Chang Lu et al. [18] proposed an acquisition strategy to calculate working resistance by subtracting the driving force with other resistance, such as friction, according to the overall force of the loader during operation. Yu Luping [19] designed a pin sensor and obtained the relationship between the pin force and output voltage on three axes through a calibration test bench. Some researchers [20] proved that the load acquisition system is credible by comparing the theoretical value and the experimental value of the external load of the working device. Lin Beiling et al. [21] proposed a method that obtains six-dimensional resistance by three-dimensional force sensors. Moreover, they built a test bench for loader working resistance to verify the accuracy of the proposed method. Similarly, Fan Dandan [22] used a similar approach when working on the design of the loader test stand. In terms of sensor arrangement, Hou Liang et al. [23], in order to select the optimal configuration with fewer sensors, introduced the Redundancy Complementariness Dispersion and Relevance (RCDR) based feature selection method.

The above studies provide a useful reference for the theoretical research, modeling and simulation, and acquisition strategy of working resistance and trajectory. However, the following limitations still exist: The existing studies mainly focus on theoretical calculation and simulation analysis. There are few studies on the acquisition strategy, the experimental data of the relevant research is only part of the core values of the working resistance and working trajectory acquisition, the final working resistance and working trajectory are not obtained, and their effectiveness needs to be studied. To overcome these limitations, this paper proposes an acquisition strategy of wheel loader working resistance and working trajectory based on operation test and simulation test verification, which provides a feasible and complete scheme for the collection of wheel loader working resistance and working trajectory.

## 2. Design of Trajectory Acquisition Strategy Based on Kinematic Analysis

The wheel loader’s working trajectory is the position and orientation of a specific point on the bucket over time. In this study, the bucket tip was chosen as the specific point Currently, the acquisition instrument of working trajectory is lacking in engineering. In addition, the shovel tip is in close contact with the shoveled material when it is working, which makes the acquisition instrument based on contact sensors fail to work. To address this issue, some acquisition strategies formulate the key parameters of the working trajectory acquisition and then calculate the working trajectory indirectly through mathematical models. For instance, Wang Xiaoming et al. [24] obtained the key parameters of the working trajectory by analyzing the kinematic model of the wheel loader’s mechanism. Zang Hongbin et al. [25] then performed a kinematic analysis of the loader working device based on the chi-square coordinate transformation and verified it with ADAMS simulation. Therefore, this paper proposes a method to perform kinematics analysis of the reverse six-bar linkage mechanism of the wheel loader’s front-end working device to extract the key parameters required by working trajectory acquisition.

### 2.1. Kinematic Analysis

**Figure 1:**
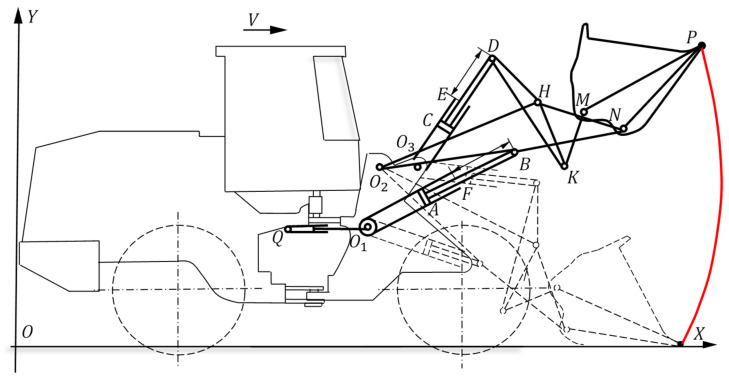
Working trajectory diagram of the loader’s shoveling process.

The movement of the bucket of the wheel loader when shoveling materials is related to the movement of two parts: one is the walking mechanism that moves the vehicle forward, and the other is the operating mechanism that drives the front-end device of the wheel loader to make a shoveling action. The working trajectory diagram of the wheel loader’s shoveling process is shown in Figure 1, which is related to the walking mechanism and operating mechanism.

When analyzing the motion relationship of the wheel loader, the D–H (Denavit–Hartenberg) coordinate system analysis method [26] is adopted. Then, by specifying the joint coordinate system, the changes between all adjacent coordinate systems are combined to determine the origin of the initial coordinate system and the total change of the end joint. Based on this, the kinematics equation can be established. T nN+1 is the transformation matrix that transforms one coordinate system to the next reference coordinate system, and its mathematical expression is defined as follows:

(1) T nN+1=An+1=Rotz,θn+1×Trans0,0,dn+1×Transan+1,0,0×Rotx,αn+1=cθn+1−sθn+1sθn+1cθn+100000         00         0100110010     00     000001dn+10    110010an+10 0 00001   0   0   1   100cθn+100  −sθn+10  0sθn+100cθn+1001=cθn+1−sθn+1cαn+1sθn+1sαn+1an+1cθn+1sθn+1cθn+1cαn+1−cθn+1sαn+1an+1sθn+10sαn+1sαn+1dn+10001

In the above formula,

*N* is the transformation matrix number after the initial and final coordinate system is selected;

*n* is the transformation matrix number when the coordinates are transformed;

θ is the rotation angle about the connecting axis of two coordinate systems;

α is the angle between two adjacent *z*-axes (joint torsion);

*d* is the distance between two adjacent common perpendiculars on the *z*-axis;

*a* is the length of each common perpendicular (joint offset);

$s\theta_{n+1}$ is the abbreviation of $sin(\theta_{n+1})$, the same as below;

$c\theta_{n+1}$ is the abbreviation of $cos(\theta_{n+1})$, the same as below.

There are three hinge points: the connection point O_1_ between the front frame and the lifting cylinder, the connection point O_2_ between the front frame and the boom, and the connection point O_3_ between the front frame and the rotary bucket cylinder. Then, taking the bucket tooth tip P as the end joint, three working spaces can be established.

**Figure 2:**
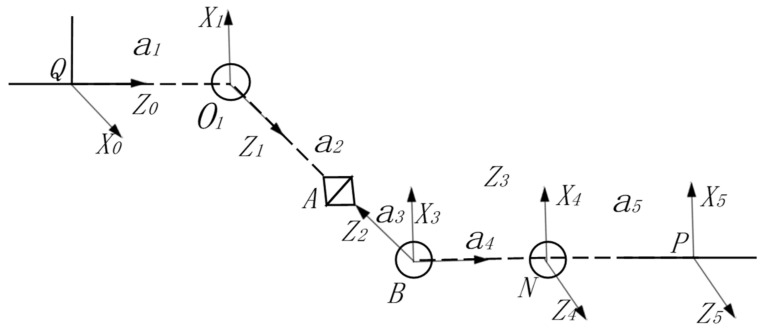
The coordinate graph based on a 3D model.

Taking the center of gravity of an unloaded loader as the center, then taking the wheel loader’s center Q to O_1_ and bucket tooth tip P at the initial position as an example, an analysis is performed on the Denavit–Hartenberg (D–H) coordinate system to simplify the complex mechanism into joints and bars and establish the coordinate diagram, as shown in Figure 2.

According to the coordinate diagram, the D–H parameters can be determined through five coordinate system transformations, and they are listed in Table 1.

By substituting the above parameters into the transformation matrices, namely, $A_{1}$, $A_{2}$, $A_{3}$, $A_{4}$ and $A_{5}$, the total transformation matrix can finally be obtained:

(2) $$\left.\begin{array}{ccc} A_{1}=\left[\begin{array}{cc} \begin{array}{cc} 1 & 0\\ 0 & 0 \end{array} & \begin{array}{cc} 0 & a_{1}\\ 1 & 0 \end{array}\\ \begin{array}{cc} 0 & 1\\ 0 & 0 \end{array} & \begin{array}{cc} 0 & 0\\ 0 & 1 \end{array} \end{array}\right] & A_{2}=\left[\begin{array}{cc} \begin{array}{cc} c_{2} & -s_{2}\\ s_{2} & 0 \end{array} & \begin{array}{cc} s_{2} & a_{2}c_{2}\\ c_{2} & a_{2}s_{2} \end{array}\\ \begin{array}{cc} 0\; & 1\\ 0\; & 0 \end{array} & \begin{array}{cc} 0\; & 0\;\\ 0\; & 1\; \end{array} \end{array}\right] & \\ A_{3}=\left[\begin{array}{cc} \begin{array}{cc} 1 & 0\\ 0 & 0 \end{array} & \begin{array}{cc} 0 & a_{3}\\ 1 & 0 \end{array}\\ \begin{array}{cc} 0 & -1\\ 0 & 0 \end{array} & \begin{array}{cc} 0\; & 0\;\\ 0\; & 1\; \end{array} \end{array}\right] & A_{4}=\left[\begin{array}{cc} \begin{array}{cc} c_{4} & -s_{4}\\ s_{4} & c_{4} \end{array} & \begin{array}{cc} 0 & a_{4}c_{4}\\ 0 & a_{4}s_{4} \end{array}\\ \begin{array}{cc} 0\; & 0\\ 0\; & 0 \end{array} & \begin{array}{cc} 1\; & 0\;\\ 0\; & 1\; \end{array} \end{array}\right] & A_{5}=\left[\begin{array}{cc} \begin{array}{cc} c_{5} & -s_{5}\\ s_{5} & c_{5} \end{array} & \begin{array}{cc} 0 & a_{5}c_{5}\\ 0 & a_{5}s_{5} \end{array}\\ \begin{array}{cc} 0\; & 0\\ 0\; & 0 \end{array} & \begin{array}{cc} 1\; & 0\;\\ 0\; & 1\; \end{array} \end{array}\right] \end{array}\right\}$$

In the above formula,

$s_{i}$ is the abbreviation of $sin(\theta_{i})$, the same as below;

$c_{i}$ is the abbreviation of $cos(\theta_{i})$, the same as below.

(3) T P1=A1A2A3A4A5=c5(c2c4-s2s4)-s5(c2s4+c4s2)-c5(c2s4+c4s2)-s5(c2c4-s2s4)-s2a1+a2c2+a3c2+a4c2c4-a4s2s4+a5c5(c2c4-s2s4)-a5s5(c2s4+c4s2)0010-c5(c2s4-c4s2)-s5(c2c4+s2s4)s5(c2s4-c4s2)-c5(c2c4+s2s4)0a2s2+a3s2-a4c2s4+a4c4s2-a5c5(c2s4-c4s2)-a5s5(c2c4+s2s4)0001

Each time the initial and final starting points are changed and the coordinate system is determined, a new coordinate sketch is needed to calculate *A*_n+1_. Similarly, the total transformation matrices of Q–O_2_–P and Q–O_3_–P can be obtained as follows:

(4) T P2=A1A2A3=c2c3 - s2s3-c2s3 - c3s2 s2a1+a2c2+a3c2c3 - a3s2s3 s3c30a3s3c3s2 - s2s3c2a2s2+a3c3s20001

(5) T P3=A1A2A3A4A5A6=c6(c5(c2c4-s2s4)-s5(c2s4+c4s2))-s6(c5(c2s4+c4s2)+s5(c2c4-s2s4))-c6(c5(c2s4+c4s2)+s5(c2c4-s2s4))-s6(c5(c2c4-s2s4)-s5(c2s4+c4s2))-s2a1+a2c2+a3c2+a4c2c4-a4s2s4+a6c6(c5(c2c4-s2s4)-s5(c2s4+c4s2))-a6s6(c5(c2s4+c4s2)+s5(c2c4-s2s4))0010-c6(c5(c2s4-c4s2)+s5(c2c4+s2s4))-s6(c5(c2c4+s2s4)-s5(c2s4-c4s2))s6(c5(c2s4-c4s2)+s5(c2c4+s2s4))-c6(c5(c2c4+s2s4)-s5(c2s4-c4s2))0a2s2+a3s2-a4c2s4+a4c4s2-a6c6(c5(c2s4-c4s2)+s5(c2c4+s2s4))-a6s6(c5(c2c4+s2s4)-s5(c2s4-c4s2))0001

It can be seen from the transformation matrix that to obtain the position of the end joint P through zero position Q, the length a of each common perpendicular and the rotation angle α should be obtained in real-time. Specifically, the value of Q can be obtained from the triangle formed by the length and elongation of the walking cylinder, lifting cylinder, and rotating bucket cylinder and the loader structure by the law of cosines, or the fixed design parameters of the loader. The value of α can be obtained by converting the length and elongation of the walking cylinder, lifting cylinder, and rotating bucket cylinder, or the length of the fixed parts of the loader.

Therefore, according to the kinematic analysis of the loader, indirect acquisition parameters related to the working trajectory can be obtained, including the displacement of the lifting cylinder, the displacement of the bucket cylinder, and the forward distance of the loader from the coordinate origin O.

### 2.2. Acquisition Strategy Design

Based on the kinematics analysis of the loading process of the wheel loader, indirect acquisition parameters related to the working trajectory are obtained. The specific acquisition scheme of the working trajectory is designed as follows. First, a laser displacement sensor is installed to measure the displacement of the lifting cylinder at the flange plate of the connection between the lifting cylinder and the lifting rod. Then, a laser displacement sensor is installed at the flange of the connection between the bucket cylinder and the bucket rod, and a reflective measuring plate is installed at the front end of the rod to measure the displacement of the bucket cylinder. Finally, the moving distance of the wheel loader is calculated by installing GPS on the top of the vehicle to obtain the real-time speed. 

## 3. Design of Resistance Acquisition Strategy Based on Statics Analysis

**Figure 3:**
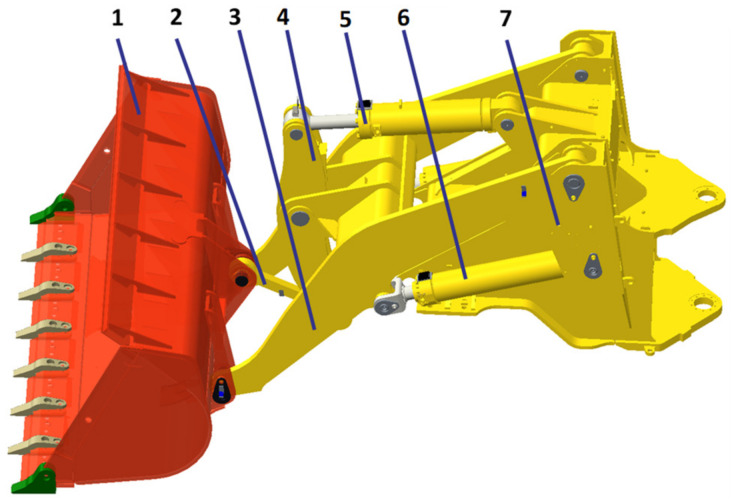
Working device structure drawing of loader: 1. bucket, 2. connecting rod, 3. boom, 4. swing arm, 5. bucket cylinder, 6. lifting cylinder, 7. front frame.

Working resistance is the external load of the material acting on the bucket when the wheel loader excavates the material. It cannot be measured directly by a single form of force sensor, and its value changes all the time in the excavation process for different excavation materials and excavation tracks. Therefore, working resistance needs to be measured indirectly. To analyze and determine the indirect parameters required for collecting working resistance, this study first conducts statics analysis on the bucket part of the wheel loader’s working device. The structure of the working device and the relationship of the components are shown in Figure 3.

By analyzing the force of the bucket under working resistance, the hinge point of indirect force acquisition that represents the working resistance of the bucket can be obtained. Then, the layout point of the force sensor is designed, and the concrete implementation scheme is formulated.

### 3.1. Statics Analysis

**Figure 4:**
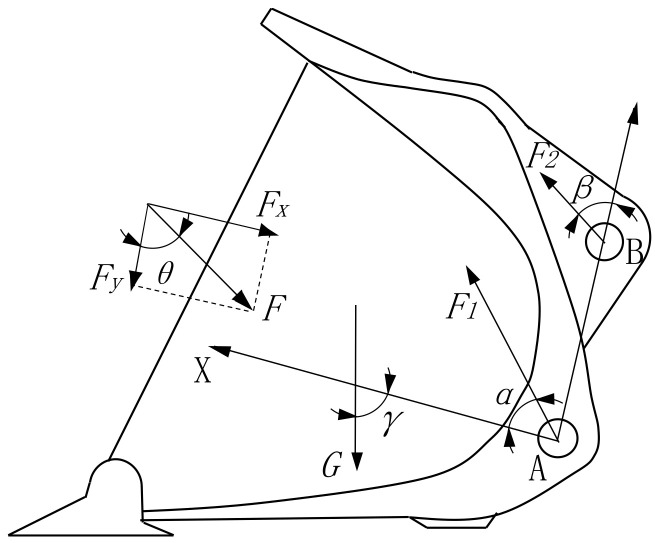
The force diagram of the left side of the bucket.

The structural stress on the left side of the loader bucket is shown in Figure 4. A plane rectangular coordinate system can be established by setting the hinge point A of the bucket and the boom as the origin. The line between the hinge point B and A of the bucket and the connecting rod is taken as the *Y*-axis, and the vertical line of the connection line between point A and point B is taken as the *X*-axis. *F* represents the working resistance of the bucket, and it is projected along the *X*-axis and *Y*-axis to obtain components *F_x_* and *F_y_*. θ is the angle between *F_y_* and *F*.

According to the statics equilibrium equation, we have:

(6) $$F_{x}=G\cos\gamma -F_{1}\cos\alpha -F_{2}\sin\beta$$

(7) $$F_{y}=G\sin\gamma -F_{1}\sin\alpha -F_{2}\cos\beta$$

According to Equations (6) and (7), the external load F can be obtained as:

(8) $$F=\sqrt{F_{\;}{}_{x}^{2}+F_{\;}{}_{y}^{2}}$$

(9) $$\theta =\tan^{-1}\frac{F_{x}}{F_{y}}$$

In the above formula, $F_{1}$ is the force at the hinge point A of the bucket and the boom; $F_{2}$ is the force at the hinge point B between the bucket and the connecting rod; G is the gravity acting on the bucket; α is the angle between the boom axis and the perpendicular of AB; β is the angle between the connecting rod axis and the hinge point AB; and γ is the remainder of the angle between the *X* direction and horizontal plane.

The statics analysis of the bucket structure indicates that the following angles are related to working resistance: the included angle α between the boom axis and the perpendicular connecting the hinge point *AB*, the angle β between the connecting rod axis and the articulated point *AB*, and the residual angle γ between the *X* direction and the horizontal plane. The forces related to working resistance include the gravity G acting on the bucket, the force $F_{1}$ at the articulated point *A* of the bucket and the boom, and the force $F_{2}$ at the articulated point *B* of the bucket and the connecting rod.

### 3.2. Simulation Verification of the Static Model

**Figure 5:**
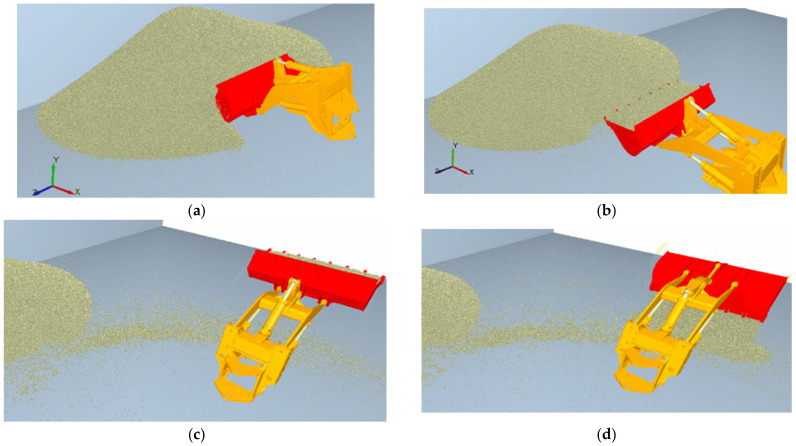
Co-simulation process with RecurDyn–EDEM: (**a**) insertion phase, (**b**) full load transport, (**c**) lifting stage, (**d**) unloading stage.

To verify the accuracy of the established mechanical model, this study used EDEM to collect the materials at the test site and model the material particles to restore the field condition of the collection. Then, RecurDyn was adopted to construct the dynamics model of the working device in the wheel loader. Based on the discrete element material model and the dynamics model, the “V”-type [27] operation cycle was simulated, as shown in Figure 5. The simulation parameter settings were obtained from [28].

Through post-processing of the RecurDyn–EDEM co-simulation results, the force $F_{1}$ at the articulated point *A*; the force $F_{2}$ at the articulated point *B* between the bucket and the connecting rod; the gravity G acting on the bucket; the included angles α,β, and γ; and the real-time working resistance *F_f_* were obtained. Finally, *F_f_* was compared with *F_j_* obtained through Equations (6)–(9), and the results are listed in Table 2.

It can be seen from Table 2 that the error of the working resistance obtained by simulation and calculation was within 1.05%, indicating that the force analysis of the bucket part was accurate.

### 3.3. Acquisition Strategy Design

Based on the indirect collection parameters related to working resistance obtained from the statics analysis of the loader bucket above, a collection scheme of working resistance was designed as follows. First, a strain sensor is attached to the shaft pin to measure the force $F_{1}$. Since the pull rod is a two-force rod, the strain gauge attached to the pull rod can measure the force $F_{2}$. Then, considering that α is the angle between the axis of the boom and the perpendicular of the articulated point AB, strain gauges need to be attached to the bucket and the boom, and the difference between the two measured values is the value of α. Moreover, considering that β is the included angle between the rod axis and the articulated line among point AB, angle sensors are pasted on the bucket and the connecting rod, and the difference between the two measured values is the value of β. Finally, γ is the residual angle of the angle between the X direction and the horizontal plane, an angle sensor is attached on the bucket, and the difference between the measured value and the initial value is the value of γ.

## 4. Acquisition Strategy Validation Based on In-Service Test and Co-Simulation

### 4.1. Acquisition Scheme and Test of Working Trajectories and Resistance

**Figure 6:**
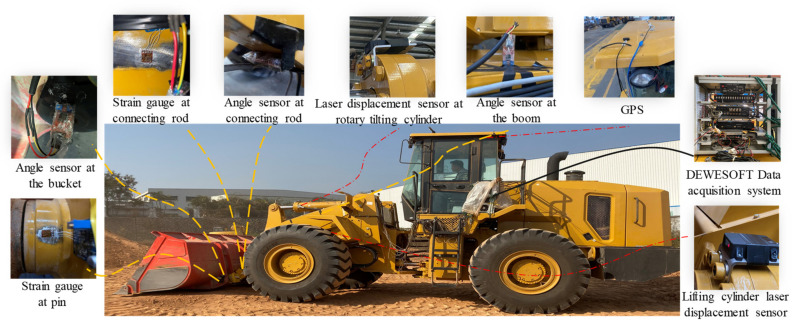
The layout of the sensors installed on the wheel loader.

To verify the effectiveness of the proposed working resistance and trajectory acquisition strategy, sensors were installed, and the sensor layout is shown in Figure 6. The indirect parameters related to working resistance and trajectory acquisition were collected.

The DEWESOFT data acquisition system in Figure 6 was used as the acquisition system in this test. The system includes a SIRIUS HD-LV module, an S-BOX data logger, and a DEWE-43 host module to meet the test requirements for acquisition of voltage, current, strain, pressure, and other types of signals. The acquisition speed is up to 200 KS/s/ch, and it has several USB ports, a CAN interface, GPS interface, WIFI module, etc. The sampling frequency of the analog-to-digital conversion was determined to be 1000 Hz by consulting the relevant literature [29,30]. In terms of the supply voltage of the acquisition system, the original battery voltage that came with the loader was converted to 24 V, 12 V, and 5 V by voltage reduction to maintain the stable operation of the acquisition data system and the multiplex sensors.

The strain gauges were BF350-3EB resistive strain gauges made of 0.02–0.05 mm diameter nickel–chromium wire, and silver-plated copper wire was used as the strain gauge lead. The angle sensor used a SCA60C single-axis tilt sensor composed of a LM393 tilt sensor chip with a rated operating voltage of 5 V, the output was the analog voltage signal corresponding to the angle conversion, and the real collected angle was 0–180°, corresponding to the voltage value of 0.5–4.5 V. Angle sensors were installed in the connecting rod, bucket, and boom parts. Laser displacement sensors used were L3s laser distance sensors with a range of 0–10 m, 1 Hz cycle measurement mode, 1 mm resolution, and 630–670 nm laser wavelength. The laser displacement sensors were installed at the flange of the lifting cylinder and on the piston rod of the lifting cylinder. The main chip selected for the GPS was an ublox UBX-M8030, which has a sensitivity tracking of −167 dBm, capturing −160 dBm and cold start −148 dBm, with an average cold start time of 24 s, and a rated voltage of 5 V or using a USB interface to provide a stable voltage supply to ensure stable reception of the signal. For better acquisition of satellite signals, the GPS needed to be installed on top of the loader to capture real-time vehicle speed signals.

The selection of the pressure sensor needed to be selected according to the pressure variation range of the object to be measured. The pressure variation range of the small chamber pressure of the lift cylinder, the small chamber pressure of the bucket cylinder, and the left steering cylinder pressure and right steering cylinder pressure was not more than 25 Mpa, so a 0–25 Mpa M14 × 1.5, FS0.2% type pressure sensor was chosen. The pressure variation range of work pump pressure, steering pump pressure, lift cylinder cavity pressure, and bucket cylinder cavity pressure did not exceed 40 Mpa, so a 0–40 Mpa M14 × 1.5, FS0.2% type pressure sensor was chosen. Finally, they were installed on the corresponding pump or cylinder components.

**Figure 7:**
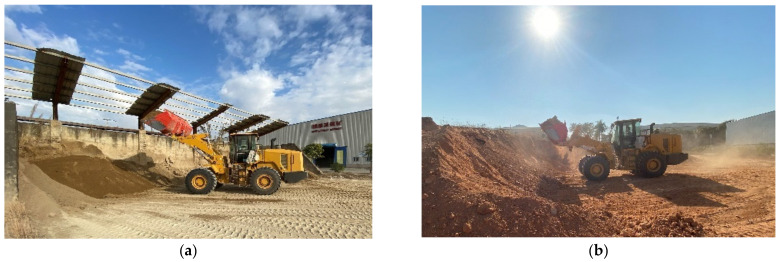
The operation site for data collection: (**a**) fine sand, (**b**) loose soil.

After completing the installation of all sensors and the construction of the data acquisition platform, all sensors needed to be commissioned first. Then the loader was required to go to the operation site to test the preliminary collected data to ensure stable operation and synchronous data collection. This study took a model XGMA wheel loader as the test object and selected fine sand and loose soil as test materials. The operation site is shown in Figure 7.

The wheel loader adopted a “V” type operation cycle mode that included the no-load forward stage, the shovel loading material stage, the full-load transportation stage, the unloading material stage, and the no-load return stage. There were 1080 operation cycles in total.

### 4.2. Indirect Measurement and Analysis of Working Trajectories

**Figure 8:**
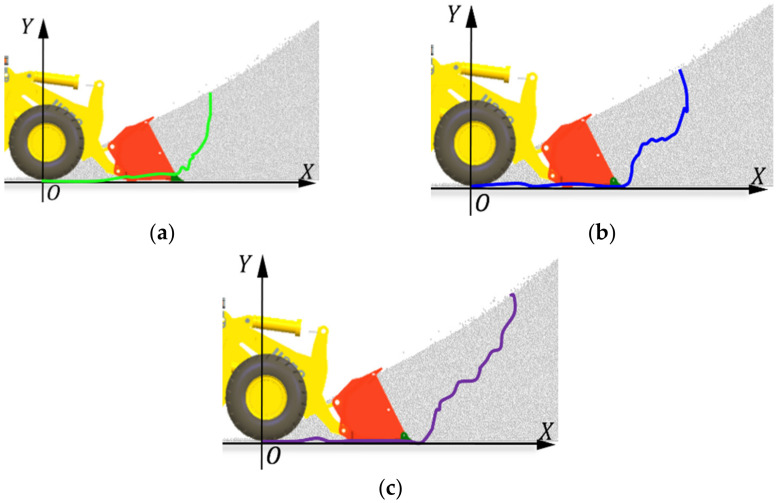
Three modes of shovel excavation: (**a**) one-time lifting from the bottom, (**b**) segmentary excavation, (**c**) empirical excavation.

To understand the trajectory changes of the shovel loading process, the test data of lifting displacement, rotating bucket displacement, and vehicle speed collected by five groups of continuous “V”-type cyclic operations were filtered and preprocessed by amplitude limiting and a new wavelet threshold [31,32]. The new wavelet threshold function [33] is shown in Equation (10). After D–H coordinate system transformation, the working trajectory of the bucket tooth tip was finally obtained and is shown in Figure 8.

(10) $${\hat{w}}_{j,k}=\left\{\begin{array}{cc} w_{j,k}(1-u)+u*sign(w_{j,k})(\left|w_{j,k}\right|-\frac{\lambda }{1+\exp({\left|\frac{w_{j,k}}{\lambda }\right|}^{n}-1)}), & \left|w_{j,k}\right|\geq \lambda \\ w_{j,k}(1-u), & \left|w_{j,k}\right|<\lambda \end{array}\right.$$

In the above formula, λ is the threshold value; *sign(.)* is the symbolic function; *u* is an adjustable coefficient within [0, 1], and it was set to 0.05 in this study; and *n* is an adjustable coefficient. Its value should be a non-zero integer and was set to 5 in this study.

**Figure 9:**
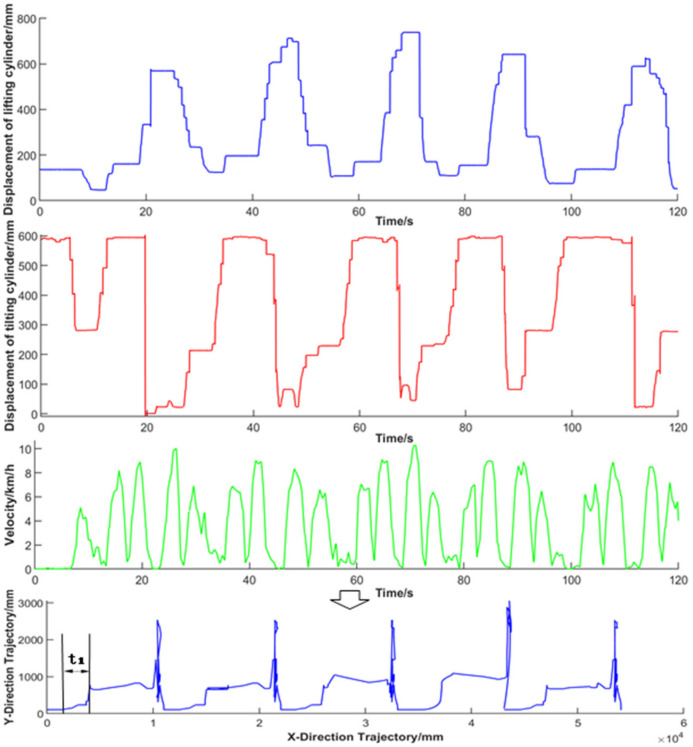
Indirect measurement parameter curve and synthetic trajectory curve.

In this test, the driver operated the wheel loader in three shovel excavation modes, including one-time lifting from the bottom, segmentary excavation, and empirical excavation. The acquired indirect measurement parameter curve and synthetic trajectory curve are shown in Figure 9, where t_1_ represents the shoveling phase of the synthetic trajectory. The one-time lifting from the bottom type was conducted to insert the bucket into the deeper part of the material and only control the bucket cylinder for one-time bucket collection; the segmentary excavation mode was conducted to insert part of the material pile after lifting by the boom and forward a section and then control the bucket cylinder to collect the bucket. The empirical excavation mode was conducted to shovel according to the driver’s experience, which consisted of multiple steps. The validity of the working trajectory acquisition strategy was verified by comparing the bucket trajectory in the shovel excavation period with that designed before the test.

### 4.3. Indirect Measurement and Analysis of Working Resistance

**Figure 10:**
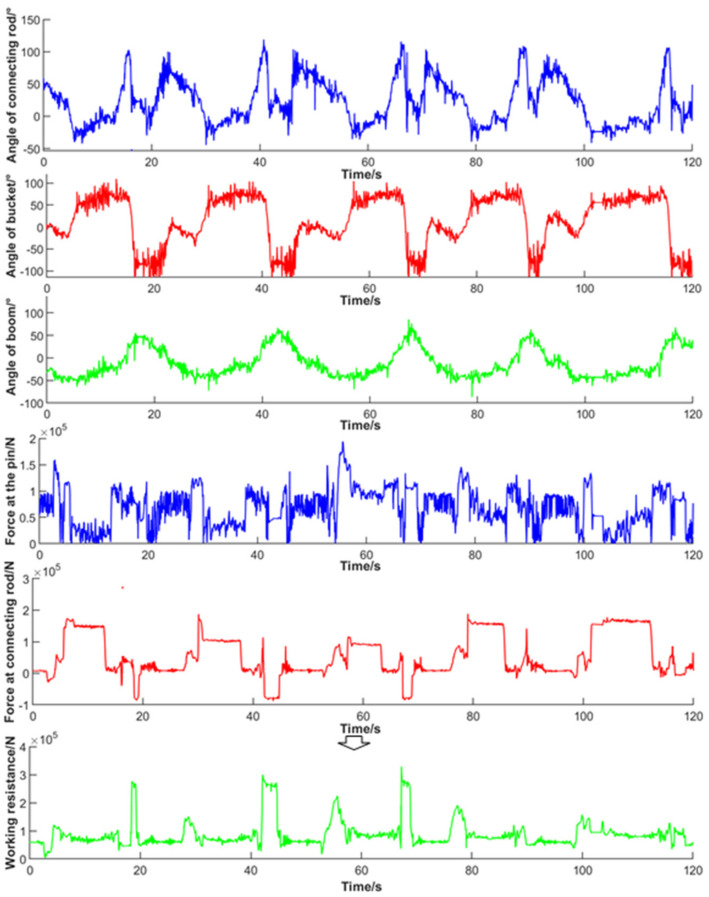
Working resistance indirect measurement parameter curve and synthetic resistance curve.

The angle acquired by five continuous groups of “V”-type cyclic operations and the pin shaft force and connecting rod force of strain conversion were processed by amplitude limiting and the new threshold of wavelet and filtered by Equations (6)–(9). Finally, the working resistance was obtained and is shown in Figure 10.

To verify the accuracy of the test data, researchers in [34,35,36] used simulation to construct a reduction test scene, and they compared the fitting degree of simulation data and test data to verify the effectiveness of the working resistance acquisition strategy.

### 4.4. Verification Analysis of RecurDyn–EDEM Co-Simulation

To verify the accuracy of the working trajectory and working resistance acquisition scheme, based on discrete element theory, a RecurDyn dynamics model of the wheel loader working device was built by considering the working condition of the EDEM collection site and the material accumulation form. During the simulation process, the “V”-type operation cycle data collected from one-time lifting from the bottom, segmentary excavation, and empirical excavation were respectively input as the trajectory, and the RecurDyn–EDEM co-simulation was carried out.

**Figure 11:**
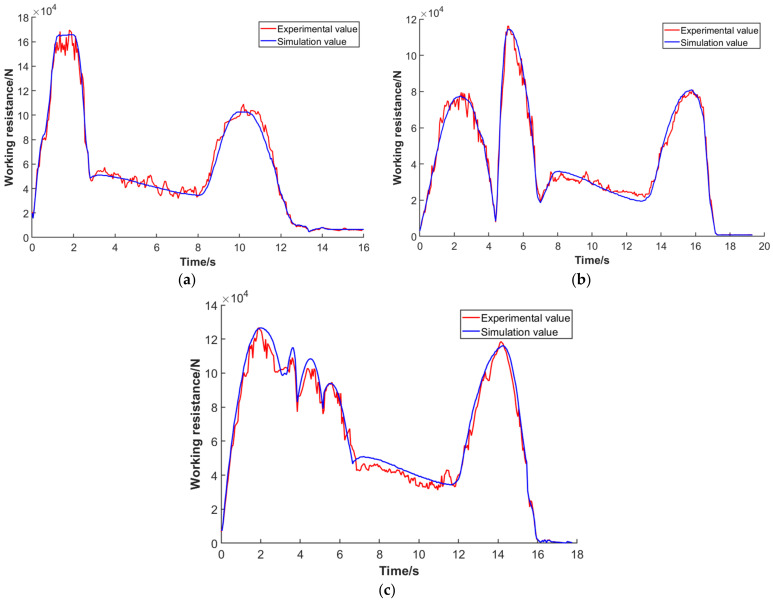
Comparison of test and simulation working resistance of the “V”-type cyclic operation in three modes: (**a**) one-time lifting from the bottom, (**b**) segmentary excavation, (**c**) driver’s experience excavation.

Based on the co-simulation results, the working resistance of the bucket during the operation cycle in the simulation process was obtained through post-processing. Then, it was compared to the collected value, as shown in Figure 11. By comparing the resistance curves under different working trajectories, it was found that when the bucket was inserted into the material pile, the number of particles inside the bucket increased, and the particles began to move, so the working resistance increased sharply. In the bucket retraction stage, under the compound movement of the lifting cylinder and rotating bucket cylinder, the bucket was retracted and gradually separated from the pile, and the working resistance was gradually reduced to less than half of the peak value. In the transportation stage, the working resistance fluctuated due to the uneven road surface but was relatively stable. In the lifting stage, the working resistance increased gradually with the lifting of the boom. In the unloading stage, the material was gradually poured out of the bucket, and the working resistance decreased and gradually approached zero.

In addition, it can be seen from the overall variation trend of the curve that the working resistance obtained by simulation was relatively smooth, while that obtained by experiment had a certain fluctuation due to the interference of external factors. To verify the correlation of the two curves, it was assumed that the simulated working resistance value was Y, and that obtained by fitting the corresponding actual test data was X. A Pearson correlation coefficient was adopted to calculate the correlation coefficient. The Pearson correlation coefficient reflected the closeness of the relationship between variables, and the value range of the Pearson correlation coefficient is between 1 and −1. The closer the data is to 0, the weaker the correlation is. When the absolute value of the Pearson coefficient is between 0 and 0.2, it is a very weak correlation or no correlation; when it is between 0.2 and 0.4, it is a weak correlation; when it is between 0.4 and 0.6, it is a moderate degree of correlation. When it is between 0.6 and 0.8, it is a strong correlation; when it is between 0.8 and 1.0, it is a very strong correlation. The formula for calculating the correlation coefficient is shown in Equation (11), where N represents the number of variable values. The calculated correlation coefficient was 0.73, which indicated a strong correlation. Thus, the working resistance curve obtained by the test was highly correlated with that obtained by simulation, showing the feasibility and correctness of the proposed working trajectory and operation acquisition scheme.

(11) $$\rho_{x,y}=\frac{\sum XY-\frac{\sum X\sum Y}{N}}{\sqrt{\left(\sum X^{2}-\frac{{\left(\sum X\right)}^{2}}{N}\right)\left(\sum Y^{2}-\frac{{\left(\sum Y\right)}^{2}}{N}\right)}}$$

**Figure 12:**
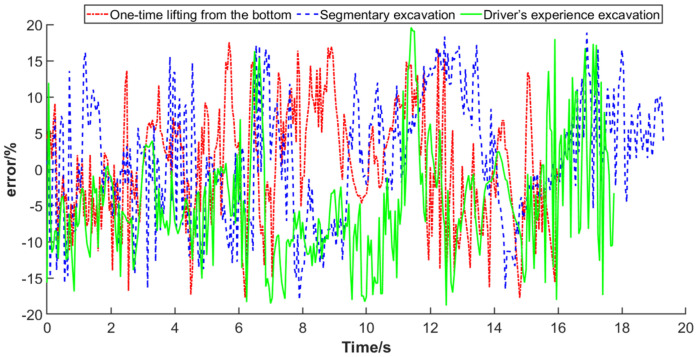
The working resistance error curves between test and simulation under different trajectories.

To further analyze the fitting degree of the working resistance curves between the test and simulation, the error values were statistically analyzed, and the error curves under different working trajectories are shown in Figure 12. Moreover, the minimum deviation, maximum deviation, and average deviation of the data in the inserting stage, bucket retraction stage, transportation stage, lifting stage, and unloading stage were obtained for comparative analysis, and the statistical results are listed in Table 3. It can be seen from Table 3 that due to the complex operating conditions of the wheel loader and many external interference factors, there was a difference between the working resistance obtained by the actual test and that obtained by simulation, and the former fluctuated greatly. For one-time lifting from the bottom, the absolute deviation ranged from 0.15% to 17.75%, with an average deviation of 6.58%; for segmentary excavation, the absolute deviation ranged from 0.07–18.87%, with an average deviation of 7%; for empirical excavation, the absolute deviation ranged from 0.05–19.59%, with an average deviation of 7.64%; for the overall data, the absolute deviation ranged from 0.05–19.59%, with an average deviation of 7.07%.

The actual working resistance is usually represented as a relative value or a trend, and it is difficult to obtain accurate real values. The correlation coefficient between the working resistance obtained by the test and simulation was 0.73, which showed a strong correlation, and the overall average deviation was 7.07%. Therefore, the feasibility of the working trajectory and working resistance acquisition scheme proposed in this paper is verified.

## 5. Discussion

This paper proposes an indirect acquisition scheme and fitting method of working resistance and working trajectory. Firstly, a static model of bucket working resistance and a kinematic model of the wheel loader’s front-end working trajectory are constructed, and the key measurement parameters of working trajectory and working resistance are analyzed and obtained. The analysis results show that the indirect measurement parameters related to the working trajectory include the displacement of the lifting cylinder, the displacement of the rotary bucket cylinder, and the forward distance of the wheel loader. The indirect measurement parameters related to the working resistance include the angle between the boom axis and the perpendicular of the articulated point AB, the angle between the connecting rod axis and the articulated point AB, the residual angle between the X direction and the horizontal plane, the bucket weight, the force at the articulated point A between the bucket and the boom, and the force at the articulated point B between the bucket and the connecting rod. Then, the corresponding working trajectory and working resistance can be fitted based on these indirect measurement parameters according to the static model and the kinematic model.

Finally, the effectiveness of the proposed acquisition scheme and fitting method is verified. The wheel loader operation test is conducted according to the acquisition scheme to obtain the key measurement parameters. Using the proposed fitting method, the corresponding test-fitted working trajectory and working resistance are obtained and analyzed. The test-fitted working trajectory is input into the RecurDyn–EDEM co-simulation model to obtain the simulation-fitted working resistance, and the correlation between the simulation-fitted working resistance and the test-fitted working resistance is analyzed.

The correlation analysis results indicate that the minimum deviation is 0.05%, the maximum deviation is 19.59%, and the average deviation is 7.07%. Due to the complex working conditions of wheel loaders and the interference of external interference factors, there are great differences between them. The actual working resistance is usually represented as a relative value or trend, and it is difficult to obtain an accurate true value. The correlation coefficient of the curves is 0.73, which shows a strong correlation. Therefore, it is proved that the wheel loader working trajectory and working resistance acquisition scheme and fitting method proposed in this paper are feasible.

## 6. Conclusions

In this paper, we propose a specific and operable indirect acquisition scheme and fitting method for working resistance and working trajectory, and we verify the feasibility by in-service testing and co-simulation. The results prove that the proposed method can be applied to practical engineering. However, the data accuracy is limited due to the restricted sensor equipment. In test data acquisition, sensor installation and debugging are not easy, and the collected data contain site operation noise. The former can be improved by optimizing sensor arrangement measurements, and the latter can be improved by enhancing filtering and denoising techniques to improve the validity of the data.

## Figures and Tables

**Table 1:** The obtained D–H parameters.

Link Variable		θ	d	a	α
1	$a_{1}$	0	0	$a_{1}$	90°
2	$\theta_{2}$	$\theta_{2}$	0	$a_{2}$	90°
3	$a_{3}$	0	0	$a_{3}$	−90°
4	$\theta_{4}$	$\theta_{4}$	0	$a_{4}$	0
5	$\theta_{5}$	$\theta_{5}$	0	$a_{5}$	0

**Table 2:** Comparison of the simulation results and the calculated values.

Operation Phase	Time (s)	*F_f_* (×10^5^ N)	*F_j_* (×10^5^ N)	Error (%)
	0.1	0.16	0.16	0
Stage of insertion	1.0	13.38	13.27	0.82
	2.0	11.71	11.62	0.77
	3.0	5.85	5.81	0.68
	4.0	5.12	5.10	0.39
Stage of transport	5.0	4.59	4.55	0.87
	6.0	4.42	4.39	0.68
	7.0	4.06	4.03	0.73
	8.0	4.42	4.42	0
Lifting stage	9.0	7.21	7.14	0.97
	10.0	11.33	11.29	0.35
	11.0	11.65	11.60	0.43
	12.0	11.17	11.13	0.36
Unloading stage	13.0	4.72	4.69	0.64
	14.0	3.25	3.22	0.92
	15.0	1.91	1.89	1.05

**Table 3:** Data comparison of working resistance between simulation and test.

Trajectory	Operation Phase	Stage of Insertion	Collecting Bucket Phase	Stage of Transport	Lifting Stage	Unloading Stage
One-time lifting from the bottom	Minimum deviation (%)	0.87	0.15	0.18	0.44	0.17
	Maximum deviation (%)	12.37	16.77	17.69	16.92	17.75
	Mean deviation	5.54	5.27	6.73	6.33	7.32
Segmentary excavation	Minimum deviation (%)	0.38	0.07	0.54	0.03	0.17
	Maximum deviation (%)	16.40	17.56	18.34	17.17	18.87
	Mean deviation	6.25	7.27	8.22	6.28	6.73
Driver’s experience excavation	Minimum deviation (%)	0.05	0.18	0.24	0.50	0.30
	Maximum deviation (%)	16.87	18.52	19.59	18.81	17.99
	Mean deviation	6.66	7.88	9.77	5.20	7.75
